# PSMA Expression in Glioblastoma as a Basis for Theranostic Approaches: A Retrospective, Correlational Panel Study Including Immunohistochemistry, Clinical Parameters and PET Imaging

**DOI:** 10.3389/fonc.2021.646387

**Published:** 2021-03-30

**Authors:** Adrien Holzgreve, Annamaria Biczok, Viktoria C. Ruf, Friederike Liesche-Starnecker, Katja Steiger, Maximilian A. Kirchner, Marcus Unterrainer, Lena Mittlmeier, Jochen Herms, Jürgen Schlegel, Peter Bartenstein, Jörg-Christian Tonn, Nathalie L. Albert, Bogdana Suchorska

**Affiliations:** ^1^Department of Nuclear Medicine, University Hospital, LMU Munich, Munich, Germany; ^2^Department of Neurosurgery, University Hospital, LMU Munich, Munich, Germany; ^3^Department of Neuropathology, University of Munich (LMU), Munich, Germany; ^4^Institute of Pathology, TUM School of Medicine, Technical University of Munich, Munich, Germany; ^5^Department of Radiology, University Hospital, LMU Munich, Munich, Germany

**Keywords:** glioblastoma (GBM), glioma, prostate specific membrane antigen (PSMA), positron emission tomography (PET), immunohistochemistry (IHC), theranostic, radionuclide therapy (PSMA-RLT), prognosis

## Abstract

**Aim:**

The aim of the current study was to enlighten the evolution of prostate-specific membrane antigen (PSMA) expression in glioblastoma between initial diagnosis and recurrence in order to provide preliminary insight for further clinical investigations into innovative PSMA-directed treatment concepts in neuro-oncology.

**Methods:**

Patients who underwent resection for de-novo glioblastoma (GBM) and had a re-resection in case of a recurrent tumor following radiochemotherapy and subsequent chemotherapy were included (n = 16). Histological and immunohistochemical stainings were performed at initial diagnosis and at recurrence (n = 96 tissue specimens). Levels of PSMA expression both in endothelial and non-endothelial cells as well as vascular density (CD34) were quantified *via* immunohistochemistry and changes between initial diagnosis and recurrence were determined. Immunohistochemical findings were correlated with survival and established clinical parameters.

**Results:**

PSMA expression was found to be present in all GBM tissue samples at initial diagnosis as well as in all but one case of recurrent tumor samples. The level of PSMA expression in glioblastoma varied inter-individually both in endothelial and non-endothelial cells. Likewise, the temporal evolution of PSMA expression highly varied in between patients. The level of vascular PSMA expression at recurrence and its change between initial diagnosis and recurrence was associated with post recurrence survival time: Patients with high vascular PSMA expression at recurrence as well as patients with increasing PSMA expression throughout the disease course survived shorter than patients with low vascular PSMA expression or decreasing vascular PSMA expression. There was no significant correlation of PSMA expression with MGMT promoter methylation status or Ki-67 labelling index.

**Conclusion:**

PSMA is expressed in glioblastoma both at initial diagnosis and at recurrence. High vascular PSMA expression at recurrence seems to be a negative prognostic marker. Thus, PSMA expression in GBM might present a promising target for theranostic approaches in recurrent glioblastoma. Especially PSMA PET imaging and PSMA-directed radioligand therapy warrant further studies in brain tumor patients.

## Introduction

Prostate-specific membrane antigen (PSMA) is a well characterized and validated marker for prostate cancer. PSMA is an enzyme with various physiological functions in non-tumorous tissues and synonymous to glutamate carboxypeptidase II (GCPII), N-acetyl-L-aspartyl-L-glutamate peptidase I (NAALADase I), NAAG peptidase and folate hydrolase 1 (FOLH1) (1–5). In oncology, PSMA has been studied for more than thirty years and is recognized as a valuable functional target for cancer therapy (6, 7). Admittedly, its naming is misleading, since PSMA expression is not only restricted to carcinomas of the prostate alone: Early reports have already shown that some tissues such as salivary glands have a physiological expression of PSMA and that monoclonal antibodies to the extracellular domain of PSMA also strongly react with the tumor vasculature in a variety of other cancers including lung, breast and colon carcinoma (8–10). However, malignant central nervous system tumors have not been studied in those early investigations on PSMA expression in non-prostatic cancers – at first, only a single case of a glioblastoma patient had been analyzed and the tumor was found to express PSMA on its neo-vasculature as well (11). Meanwhile, several immunohistopathological studies confirmed PSMA expression in the tumoral vessels of high grade gliomas (12–16).

However, it still remains unclear whether PSMA is expressed both at initial diagnosis and at tumor recurrence and if the PSMA expression is altered under multimodal therapy (17, 18). These issues are all the more of particular interest given the current rise of available PSMA-directed theranostic concepts, which could be implemented in the management of recurrent glioblastoma as an innovative therapeutic approach. Especially in prostate cancer, where PSMA is highly expressed at recurrence as well, PSMA-targeted positron emission tomography (PET) and PSMA-targeted radionuclide therapy constitute a main driver of current improvement of therapy management and it might be worth to implement those promising approaches in neuro-oncology as well (19–22). The dismal prognosis of glioblastoma patients underlines the need of such innovative therapies and the molecular heterogeneity of tumors classified as glioblastoma make this entity an ideal candidate for individualized and theranostic treatment concepts (23–25). First small-number case series have shown overall promising results regarding the feasibility of PSMA PET imaging in glioma patients and warrant further research in the field (26).

Therefore, we aimed to fill the knowledge gap on the temporal evolution of PSMA expression and its potential alterations under adjuvant therapy in glioblastoma in order to provide a solid basis for the implementation of innovative PSMA-directed treatment concepts in neuro-oncology. We here present a comprehensive immunohistochemical analysis of tumoral PSMA expression in a strictly selected cohort of glioblastoma patients both at initial diagnosis and at recurrence. Additionally, we demonstrate *pars pro toto* the feasibility of PSMA PET imaging after multimodal therapy in a glioblastoma patient at recurrence.

## Materials and Methods

For the current study, patients who underwent resection for de-novo glioblastoma between Dec 2007 and Nov 2015 and had a re-resection in case of a recurrent tumor following completion of radiochemotherapy and subsequent chemotherapy (at least one cycle) according to the EORTC/NCIC protocol were selected. Time of last follow up was January 1^st^ 2020. Hematoxylin and eosin (H&E) as well as PSMA and CD34 immunohistochemical stainings were performed for every patient both at initial diagnosis and at recurrence. Immunohistochemical expression of PSMA and vascular density (using CD34 staining) were quantified and changes between initial diagnosis and recurrence were determined. PSMA expression was correlated with survival time as well as established clinical parameters. As a proof-of-principle, a ^68^Ga-PSMA PET scan was obtained in one patient at the time of tumor recurrence.

### Patient Characteristics

16 patients in whom tissue from first and second surgery was available for PSMA and CD34 staining were included. Patient characteristics including sex, age, Karnofsky performance status scale (KPS) and survival data as well as tumor characteristics including *IDH* mutation status, MGMT promotor methylation status and Ki 67 labeling index if available are presented in **Table 1**. The therapeutic decision making was based on suggestions of the interdisciplinary neuro-oncological tumor board at the University Hospital of the Ludwig-Maximilians-University Munich; treatment decisions were not affected by this retrospective study. The study protocol was approved by the local ethics committee (#20-535).

**Table 1:** Patient Characteristics.

Patient ID	Sex	KPS [%]	Age [years]		Time to recurrence [months]	IDH mutation status	MGMT promotor methylation status	Ki-67 index [%]		All: Radiotherapy with concomitant TMZ (27)	Overall survival[months]
			At initial diagonsis	At recurrence				At initial diagonsis	At recurrence	Cycles of adjuvant TMZ until 2^nd^ resection:	
**1**	m	100	30	31	5	wildtype	–	20	30	3	27.8
**2**	f	70	71	72	4	wildtype	+	10	>5	1	39.6
**3**	m	80	66	68	8	wildtype	+	10	30	6	27.7
**4**	m	80	58	59	12	wildtype	+ (+/-)	10	12	3 + ISBT	17.8
**5**	m	80	64	65	8	wildtype	–	>5	n. a.	6	17.4
**6**	m	80	42	43	7	wildtype	+	50	20	6	51.2
**7**	m	80	49	50	6	wildtype	–	20	15	4-5	20.7
**8**	f	90	56	57	7	wildtype	+	20	>5	TMZ + CCNU	70.3
**9**	f	90	41	42	15	wildtype	+	50	n. a.	2 + 6 (i)	33.0
**10**	m	100	25	25	6	mutant	+	30	n. a.	3	17.7
**11**	f	80	66	69	33	n. a.	+	> 5	18	8 (C) + 1 (i) + 6 (i) + ISBT	59.2
**12**	m	80	57	59	23	wildtype	+	25	n. a.	12 (C)	51.2
**13**	m	80	65	66	10	wildtype	–	30	80	3 + 2 (i)	13.5
**14**	f	90	44	45	13	wildtype	–	20	n. a.	11	46.2
**15**	m	80	74	74	8	wildtype	+	>5	1	3	13.2
**16**	m	90	51	52	12	wildtype	–	45	n. a.	6	31.3

m, male; f, female; +, methylated; –, unmethylated; +/−, partially methylated; n. a., not available; TMZ, temozolomide; i, intensified TMZ according to DIRECTOR (28); CCNU, lomustine [according to CeTeG (29)]; C, with additional cilengitide [according to CENTRIC (30)]; ISBT, interstitial brachytherapy.

### Histology and Immunohistochemistry

Tissue samples were first analysed according to clinical routine including hematoxylin- and eosin (H&E) staining and molecular genetic analyses (*IDH* mutation status, MGMT promoter methylation status). Two H&E-stained slides per patient (primary and recurrent tumor each) were systematically reviewed to confirm the diagnosis of glioblastoma (WHO grade IV) according to the 2016 WHO classification of tumors of the central nervous system (31) and to evaluate the extent of microvascular proliferation. Additional immunohistochemical stainings were performed on 5 µm thick formalin-fixed and paraffin-embedded (FFPE) tissue sections. For PSMA immunohistochemistry, slices were pre-treated with Ventana Cell Conditioner 1 immunostainer (Ventana Medical Systems, Oro Valley, AZ, USA) for 1 h and subsequently incubated with mouse monoclonal PSMA antibody 3E6 (1:50, Agilent Technologies, Santa Clara, CA, USA) for 32 min. For CD34 immunohistochemistry, slices were incubated with monoclonal mouse anti-CD34 antibody QBEnd10 (1:400, Agilent Technologies) without pre-treatment for 32 min. Both immunohistochemical stainings were performed on a Ventana BenchMark Ultra automated stainer with the iView DAB Kit (Ventana Medical Systems). Slides were counterstained with hematoxylin. Positive controls serves as quality assurance. All immunohistochemical stainings were scanned with a Pannoramic Midi II (3D HISTECH Ltd, Budapest, Hungary).

### Quantification of Immunohistochemistry

Several approaches were chosen to quantify the expression both of PSMA and CD34. First, the amount of PSMA-positive and CD34-positive vessels per slide was counted. Second, to account for a variable extent of tumor tissue per slide, a score ranging from 0 to 5 was assigned to the amount of PSMA-positive or respectively CD34-positive vessels per tumor area (where 0 = no tumor vessels, 1 = single tumor vessels, 2 = several tumor vessels, 3 = intermediate density of tumor vessels, 4 = high vessel density and 5 = very high vessel density). Finally, PSMA-to-CD34 ratios were calculated for the amount of vessels and for the score of vessel density. In addition to the vascular PSMA expression, the immunoreactive score (IRS) for PSMA expression was assessed in non-endothelial cells within the tumor. The IRS is the product of a score for the intensity of PSMA-staining (ranging from 0 to 3; where 0 = no positive reaction, 1 = weak intensity, 2 = moderate intensity, 3 = strong intensity) and the amount of PSMA-positive cells (ranging from 0 to 4; where 0 = no positive cells, 1 = less than 10% positive cells, 2 = 10-50% positive cells, 3 = 51-80% positive cells and 4 = more than 80% positive cells) as established by Remmele and Stegner (32). Both fully manual and (semi-)automatic quantification approaches were tested using QuPath (33). A fully automatic quantification algorithm had to be discarded because of a lack of reliable differentiation between endothelial and non-endothelial PSMA staining.

### Statistical Analysis

Wilcoxon signed-rank test was used for comparison of means. Correlation of PSMA expression with clinical parameters was tested at primary diagnosis and at recurrence using a two way analysis of variance (ANOVA). Kaplan-Meier estimator, Log-rank test and Cox proportional hazards model were used for survival analyses. Overall survival time was defined as time from first tumor surgery at initial diagnosis until date of last follow up. Post-recurrence survival time was defined as time from second tumor surgery at first recurrence until date of last follow up. Significance level was set at p<0.05.

### PSMA Positron Emission Tomography (PET)

Patient #16 received a gallium-68 [^68^Ga]-PSMA-11 PET scan at recurrence. The PET scan was performed as previously described (34). 141 MBq ^68^Ga-PSMA-11 were intravenously injected. The acquisition time was 60–80 min post-tracer injection. A low dose computed tomography (ldCT) scan was performed for attenuation correction.

## Results

### PSMA and CD34 Immunohistochemistry at Initial Diagnosis and at Recurrence

The original numeric data from all immunohistochemical analyses are displayed in **Table 2**.

**Table 2:** Original numeric data from all immunohistochemical analyses.

Patient ID	1^st^ surgery at initial diagnosis							2^nd^ surgery at recurrence						
	PSMA-positive vessels		PSMA-positive non-endothelial cells			CD34-positive vessels		PSMA-positive vessels		PSMA-positive non-endothelial cells			CD34-positive vessels	
	amount	score	intensity	amount	IRS	amount	score	amount	score	intensity	amount	IRS	amount	score
**1**	368	4	1	2	2	763	4	252	4	3	3	9	265	5
**2**	28	1	1	1	1	231	2	8	1	2	1	2	187	3
**3**	83	2	2	1	2	294	2	135	3	1	1	1	212	3
**4**	437	4	1	1	1	503	4	3	1	1	3	3	38	1
**5**	102	2	2	2	4	1437	4	75	2	2	2	4	208	4
**6**	32	1	1	1	1	83	2	72	4	2	2	4	243	3
**7**	52	3	2	2	4	152	2	26	1	3	4	12	53	2
**8**	379	4	2	1	2	635	5	3	4	1	2	2	55	2
**9**	203	4	1	2	2	n. a.	5	99	2	2	2	4	220	3
**10**	42	1	1	1	1	245	2	57	2	1	2	2	323	4
**11**	91	2	2	1	2	193	2	8	1	2	3	6	98	2
**12**	7	1	2	2	4	172	3	0	0	0	0	0	57	3
**13**	66	2	1	1	1	137	3	283	3	0	0	0	436	4
**14**	54	2	0	0	0	233	4	37	2	2	1	2	68	2
**15**	376	4	2	2	4	1080	5	31	2	3	2	6	310	4
**16**	205	3	0	0	0	338	3	185	3	3	1	3	498	5

IRS, immunoreactive score; n. a., not available.

The evolution of immunohistochemical parameters between first and second surgery is displayed in **Table 3**.

**Table 3:** Evolution of immunohistochemical parameters over time.

Patient ID	Changes between initial diagnosis and recurrence									Post recurrence survival [months]
	PSMA-positive vessels		CD34-positive vessels		PSMA/CD34ratios		PSMA-positive non-endothelial cells			
	amount	score	amount	score	amount	score	intensity	amount	IRS	
**1**	-32%	↔	-65%	↑	↑	↓	↑	↑	↑	22.2
**2**	-71%	↔	-19%	↑	↓	↓	↑	↔	↑	35.3
**3**	+63%	↑	-28%	↑	↑	↔	↓	↔	↓	7.5
**4**	-99%	↓	-92%	↓	↓	↔	↔	↑	↑	5.6
**5**	-27%	↔	-86%	↔	↑	↔	↔	↔	↔	8.7
**6**	+125%	↑	+193%	↑	↓	↑	↑	↑	↑	32.4
**7**	-50%	↓	-65%	↔	↓	↓	↑	↑	↑	12.7
**8**	-99%	↔	-91%	↓	↓	↑	↓	↑	↔	63.5
**9**	-51%	↓	n. a.	↓	n. a.	↓	↑	↔	↑	18.4
**10**	+36%	↑	-32%	↑	↑	↔	↔	↑	↑	12.0
**11**	-91%	↓	-49%	↔	↓	↓	↔	↑	↑	26.8
**12**	-100%	↓	-67%	↔	↓	↓	↓	↓	↓	28.3
**13**	+329%	↑	+218%	↑	↑	↑	↓	↓	↓	3.4
**14**	-31%	↔	-71%	↓	↑	↓	↑	↑	↑	32.7
**15**	-92%	↓	-71%	↓	↓	↓	↑	↔	↑	4.5
**16**	-10%	↔	+47%	↑	↓	↓	↑	↑	↑	18.5

↔, stable; ↑, increase; ↓, decrease; IRS, immunoreactive score; n. a., not available (excluded due to unspecific CD34 staining).

**Figure 1:**
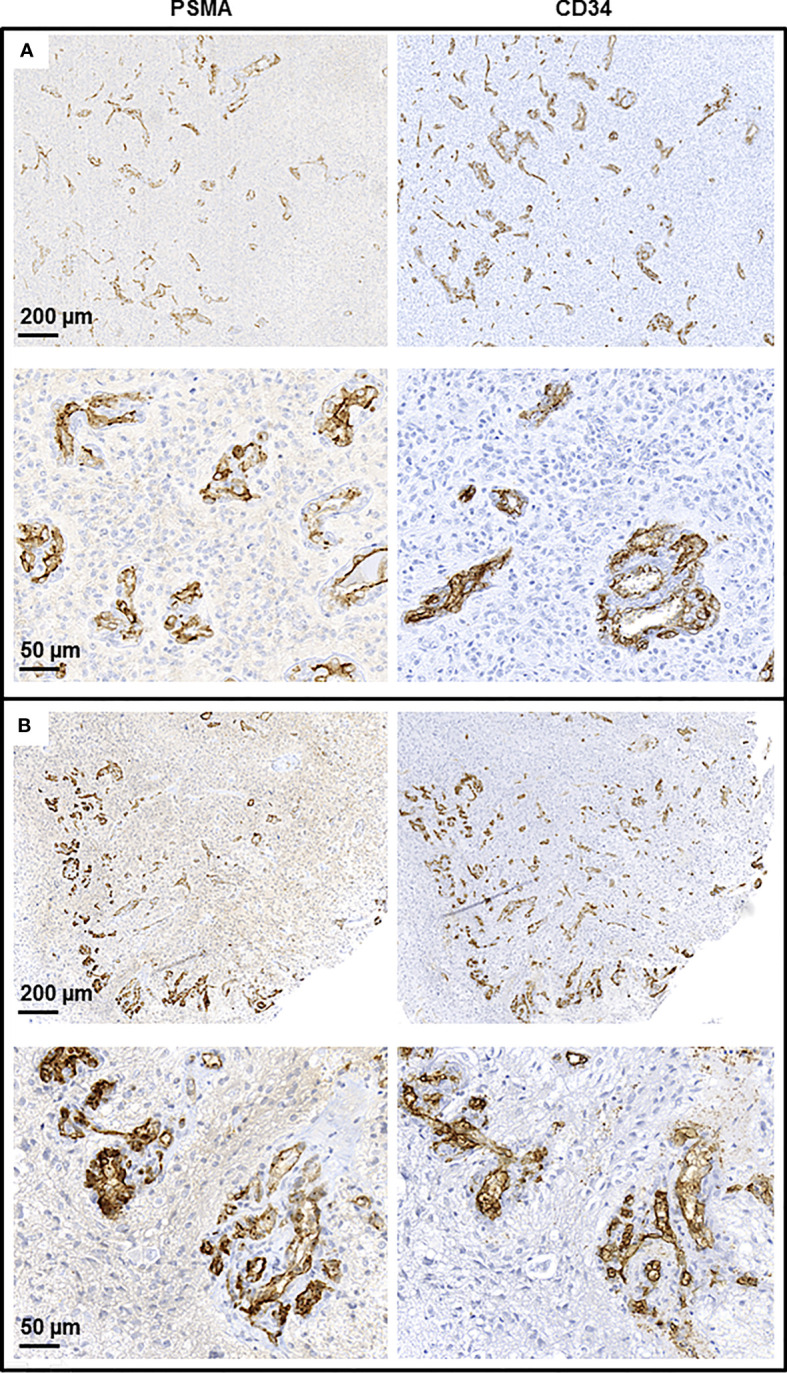
Endothelial PSMA expression in glioblastoma. Immunohistochemical staining of resected tumor tissue of an exemplary glioblastoma patient at initial diagnosis **(A)** and at recurrence **(B)**.

In brief, at initial diagnosis all 16 patients showed PSMA expression on tumor vessels, however, at a highly variable degree (median amount of counted PSMA-positive tumor vessels per tissue section 87; range 7-497). At recurrence, 15/16 patients showed PSMA expression on the tumor microvasculature; the median amount of counted PSMA-positive tumor vessels per tissue section was lower than at initial diagnosis when comparing the entire group of patients (47 vs. 87; p = 0.032). But again, the individual level of vascular PSMA expression varied considerably and ranged between 0 and 283. In contrast to the absolute number of counted vessels per tissue section, the median PSMA score remained stable and was 2 both at initial diagnosis (range 1 to 5) and at recurrence (range 0 to 4). However, there was again a high variability at the individual level: The score of PSMA-positive vessels increased in 4/16 patients, decreased in 6/16 patients and remained stable in 6/16 patients. In analogy to the PSMA findings, the median amount of CD34-positive vessels was lower at recurrence compared to initial diagnosis (210 vs. 245; p = 0.031) but the CD34 score did not significantly change at recurrence compared to initial diagnosis when considering the entire group of patients. **Figure 1** illustrates the vascular PSMA expression in glioblastoma at initial diagnosis and at recurrence.

**Figure 2:**
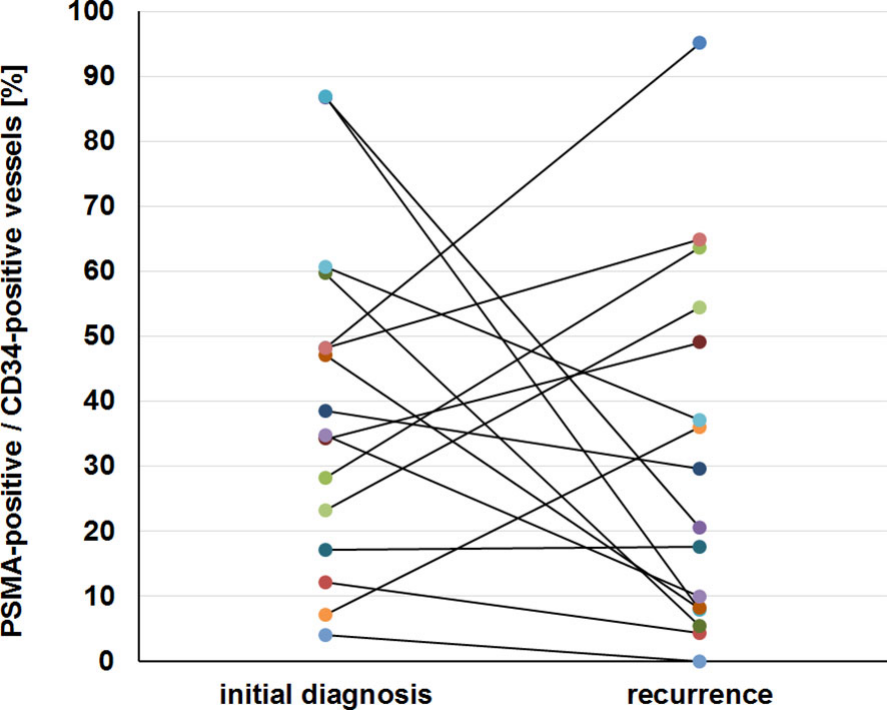
Heterogeneous PSMA expression in glioblastoma. The level of vascular PSMA expression corrected for the vessel density and its evolution from initial diagnosis to recurrence are displayed for all 16 patients included in the study.

Essentially, the proportion of PSMA-positive vessels to all CD34-positive vessels showed a wide inter-individual range both at initial diagnosis and at recurrence and it did not remain stable in the individual patient. The change of the proportion of PSMA-positive vessels between initial diagnosis and recurrence inter-individually ranged from –100% to +329% and is shown in **Figure 2**.

With very few isolated exceptions, all non-tumorous vessels in the surrounding tissues were PSMA-negative.

**Figure 3:**
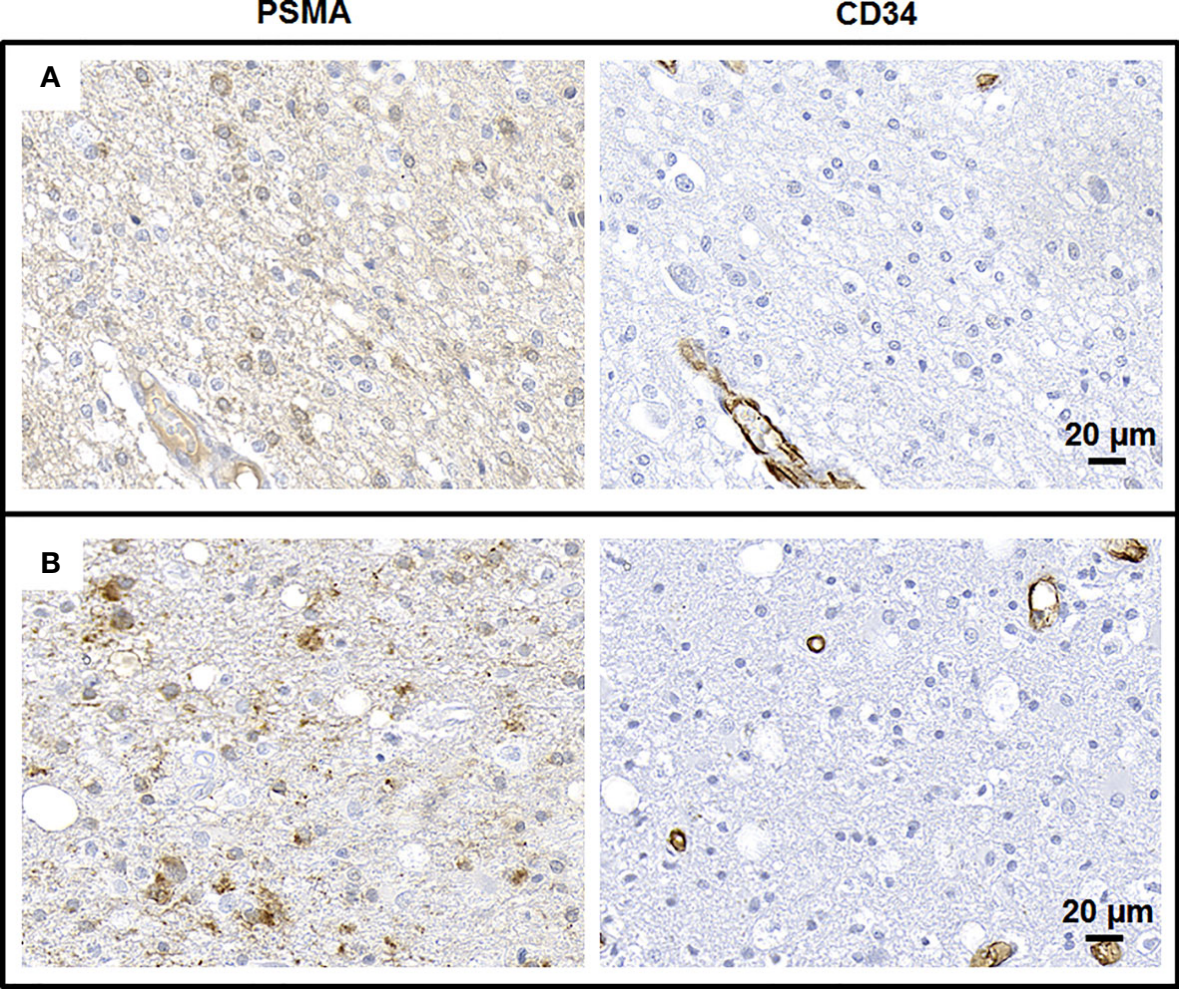
Non-endothelial PSMA expression in glioblastoma. Immunohistochemical staining of resected tumor tissue of an exemplary glioblastoma patient at initial diagnosis **(A)** and at recurrence **(B)**.

The median IRS for tumoral PSMA expression in non-endothelial cells increased from 2 at initial diagnosis to 3 at recurrence (p = 0.021). However, there was again a high inter-individual variability of PSMA expression; the IRS at initial diagnosis ranged from 0 to 6 and at recurrence from 0 to 12. **Figure 3** illustrates the non-endothelial PSMA expression in glioblastoma at initial diagnosis and at recurrence.

### Correlation of PSMA Expression With Patient Survival

The median overall survival time was 29.8 (CI 95% 18.1 – 51.8) months. The median post recurrence survival time was 18.8 (CI 95% 7.6 – 32.9) months. 8 patients experienced a second tumor recurrence and 12 patients died.

**Figure 4:**
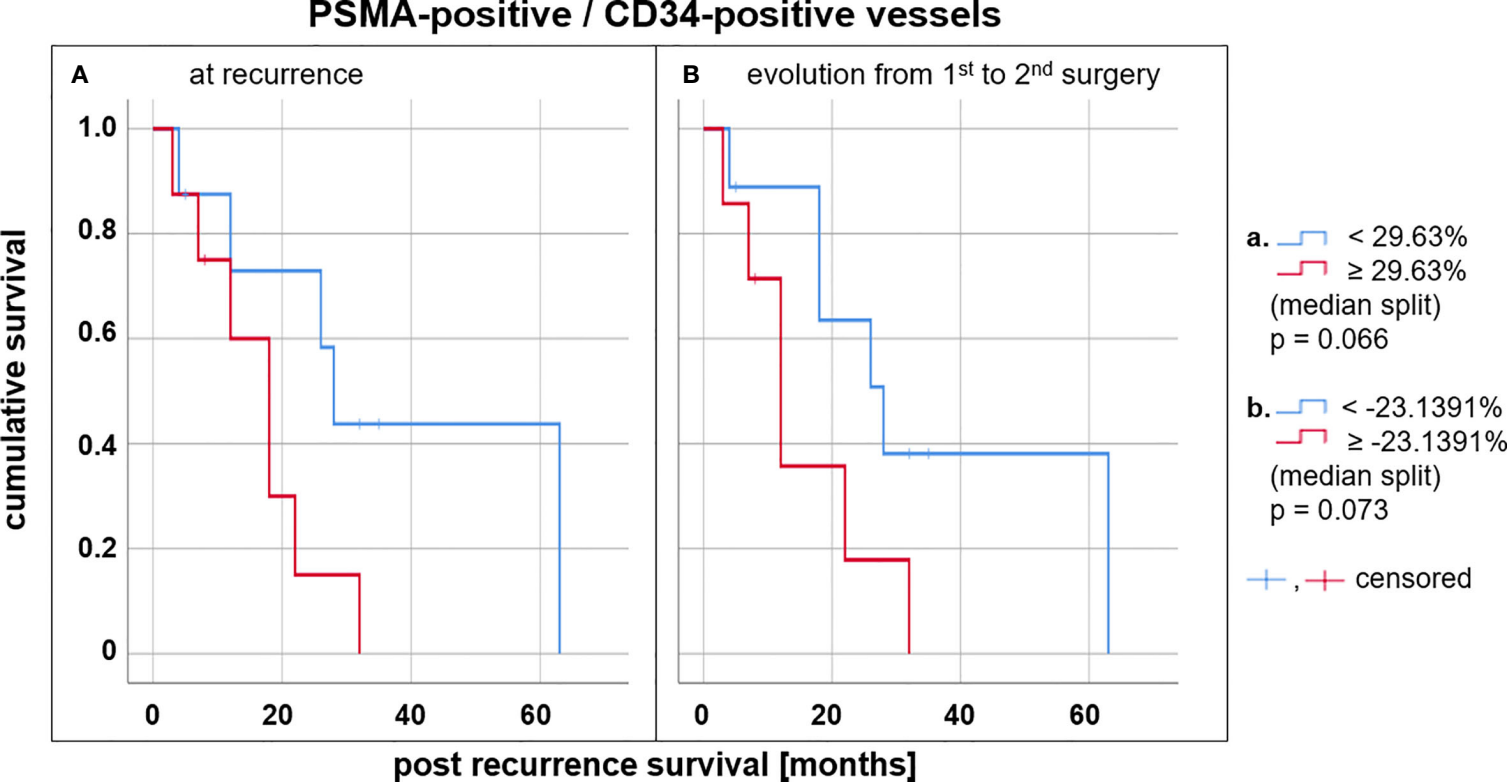
Survival analysis in glioblastoma patients depending on tumoral PSMA expression. Kaplan-Meier estimates for glioblastoma patients with a high (red) and a low (blue) vascular PSMA expression in the tumor at recurrence **(A)**. Kaplan-Meier estimates for glioblastoma patients with increasing (red) and decreasing (blue) vascular PSMA expression between primary diagnosis and recurrence **(B)**.

The level of vascular PSMA expression at recurrence (i. e. the proportion of PSMA-positive to all CD34-positive vessels) was inversely correlated with post recurrence survival time (see **Figure 4A**, median split, p = 0.066), while no such correlation could be found for PSMA expression at initial diagnosis. Furthermore, the evolution of vascular PSMA expression between primary diagnosis and recurrence was inversely correlated with post recurrence survival time: Patients with a decrease of vascular PSMA expression of more than 23.14% had a median post recurrence survival time of 22 months compared to only 12 months in patients with less decrease or an increase of vascular PSMA expression (see **Figure 4B**, median split, p = 0.073).

### Correlation of PSMA Expression With Clinical Parameters Other Than Survival

ANOVA showed no correlation of the level of vascular PSMA expression (i. e. the proportion of PSMA-positive to all CD34-positive vessels) with the MGMT promotor methylation status or with the Ki 67 labelling index, neither at initial diagnosis nor at recurrence.

### PSMA Positron Emission Tomography (PET)

**Figure 5:**
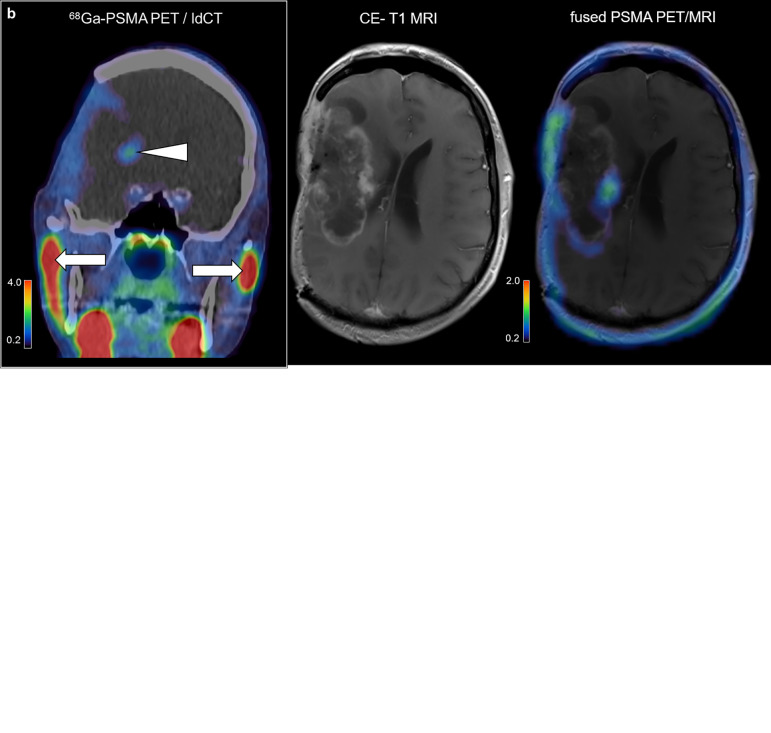
PSMA PET imaging in glioblastoma. ^68^Ga-PSMA PET scan and structural imaging modalities of an exemplary glioblastoma patient after tumor recurrence in axial plane **(A)** and coronal plane **(B)**. Arrowhead, tumoral PSMA uptake; arrows, physiological PSMA uptake in the salivary glands.

The PSMA PET scan of patient #16 after recurrence is shown in **Figure 5**. This tumor shows a predominantly low and focally moderate [^68^Ga]-PSMA-11 uptake in the area of contrast-enhancement in magnetic resonance imaging (MRI). Although the tumor-to-background contrast is extremely high (maximal tumor-to-background ratio 21.6) due to the very low [^68^Ga]-PSMA-11 uptake in the contralateral cerebral background (see **Figure 5A**), the intra-tumoral [^68^Ga]-PSMA-11 uptake is low compared to the maximal physiological [^68^Ga]-PSMA-11 uptake in the salivary glands with a ratio of 0.62 (see **Figure 5B**), which ultimately led to a discard of a PSMA radionuclide therapy in this patient.

## Discussion

The current study yields new insights on PSMA expression in glioblastoma and therefore provides the underpinning for further clinical investigations on PSMA-directed theranostic approaches in glioblastoma patients. We demonstrate in a homogeneously treated glioblastoma patient cohort that PSMA is expressed both at initial diagnosis and at recurrence after multimodal therapy, however, to a variable degree. PSMA is primarily expressed in the neo-angiogenetic tumor microvasculature but also on non-endothelial cells to some extent. Apart from supporting PSMA-directed theranostic approaches for the management of glioblastoma patients, our data suggest that PSMA expression at tumor recurrence might be of prognostic significance for the disease course.

The first report of PSMA expression in the neovasculature of glioblastoma (11) has in the meanwhile been verified in two consecutive immunohistopathological studies with together 37 glioblastoma patients (WHO° IV): All glioblastomas showed a generally high staining intensity for PSMA in their vessels (13, 15). A third study showed some PSMA immunoreactivity in brain tumor cells in three out of 52 glioblastoma patients (the vascular PSMA expression has not been investigated in those tumors) (12). A recent study found PSMA-positive staining in eleven out of 27 WHO grade IV gliomas, however without clear specification which cells expressed the PSMA (14). The most recent histopathological study on PSMA in glioblastoma included 60 patients. Most specimens showed a considerable PSMA expression of the tumor vasculature, however primary and recurrent disease was not differentiated (16). In the present study, we analyzed both PSMA expression and tumor vascularity in a strictly selected cohort of glioblastoma patients undergoing resection at initial diagnosis and re-resection at recurrence, taking account both for endothelial and non-endothelial PSMA expression. Essentially, all glioblastoma tissue seems to express PSMA to some extent both at primary diagnosis and at recurrence after multimodal therapy. Interestingly, the absolute amount of vessels (evaluated *via* CD34 staining) as well as the absolute amount of PSMA-positive vessels significantly decrease after multimodal therapy, whereas the median CD34 score and the median PSMA score remain stable, indicating that less vital tumor tissue might be present in the recurrent situation compared to initial diagnosis. It has already been shown that, especially in the recurrent situation, obtaining sufficient tissue can be challenging and that it is an important prerequisite for conclusive histopathological or further genomic diagnostics (35, 36). The chosen study design with the inclusion of only patients with open resection both in the primary situation and at recurrence takes this knowledge into account (37). To a fewer degree as compared to vessels, PSMA is expressed on non-endothelial cells as well; in the overall group the non-endothelial PSMA staining within the tumor slightly increases between initial diagnosis and recurrence in glioblastoma. According to previous immunohistochemical studies on PSMA expression in glioblastoma, the non-endothelial PSMA staining seems to be primarily related to tumor cells (12). Interestingly, preclinical and clinical data reveal PSMA radioligand uptake in activated astrocytes as well, which might confound the evaluation of PSMA uptake in non-endothelial tumor cells (38, 39). However, a reliable differentiation between tumor cells and stationary astrocytes in *IDH*-wildtype gliomas is not yet feasible using immunohistochemical methods, as there is still no marker that clearly distinguishes between both. Therefore, we cannot determine with certainty to which cell populations the non-endothelial PSMA expression in the present study can be attributed. Eventually, we show that the temporal evolution of PSMA expression in glioblastoma is a mixed picture, which underlines the advantage of individual assessment of PSMA expression *via* non-invasive PSMA PET imaging.

Several pilot studies with each a few patients included have demonstrated the general feasibility of non-invasive assessment of PSMA expression in glioblastoma *via* PET imaging (34, 40–43). Whereas those case series have used either ^68^Ga-labeled PSMA tracers such as ^68^Ga-PSMA-11 or less common PSMA-directed tracers such as ^18^F-DCFPyL or ^89^Zr-Df-IAB2M, a recent case report for the first time demonstrated the feasibility of ^18^F-labeled PSMA PET imaging in a glioblastoma patient (44). Several advantages of ^18^F-labeled compounds, mainly that no in-house ^68^Ga generator is required, have started an incipient shift from ^68^Ga- to ^18^F-labeled PSMA-targeted tracers in PET imaging of prostate cancer as well (45). Those recent developments pave the way for a potential wide availability and straightforward implementation of PSMA PET imaging in neuro-oncology. Our data support the general feasibility of PSMA PET imaging in glioblastoma by immunohistochemically confirming PSMA expression both at initial diagnosis and at tumor recurrence as well and by adding a further case of PSMA PET imaging in a glioblastoma patient to the few published reports. The most intriguing perspective of PSMA expression in glioblastoma lies in the concept of PSMA-targeted theranostics. PSMA radioligand therapy yields highly satisfactory results in patients with metastatic castration-resistant prostate cancer owing to its high response rates, low toxic effects, and reduction of pain (46) and a pivotal dosimetry case study of ^177^Lu-PSMA-617 treatment in a glioblastoma patient has shown promising preliminary results for the implementation of PSMA radioligand therapy in neuro-oncology as well (47).

Another intriguing finding is that the level of PSMA expression in glioblastoma might be a prognostic marker. According to our data, patients with a high level of vascular PSMA expression at recurrence lived shorter compared to those with a low PSMA expression. Data obtained from other neoplastic diseases show conflicting information on a possible association between tumoral PSMA expression and survival: Whereas in primary lung tumors such as non-small cell lung cancer, PSMA expression and outcome seem not to be correlated (48, 49), PSMA expression has shown to be a prognostic marker also in castration-resistant prostate cancer, adrenocortical tumors, or hepatocellular carcinoma. In those tumors, high PSMA expression correlates with a similarly poor prognosis in analogy to our data for glioblastoma patients in the present study (50–52). Interestingly, the change of the level of vascular PSMA expression between initial diagnosis and recurrence seems to be prognostic as well in glioblastoma: Patients with a strongly decreasing vascular PSMA expression survived longer than those with increasing vascular PSMA expression (22 months vs. 12 months post recurrence survival, see **Figure 4B**), which is especially interesting with regard to potential repeated non-invasive PSMA PET imaging over the disease course. For instance, patient #11 and patient #13 show a comparable level of vascular PSMA expression at initial diagnosis (proportion of PSMA-positive to all CD34-positive vessels of 47.15% and 48.18%, respectively, see **Table 2**). The level of vascular PSMA expression of patient #11 decreased by over 80% and the post recurrence survival time was 26.8 months. In patient #13 however, the vascular PSMA expression increased by over 30% which resulted in a short post recurrence survival time of only 3.4 months (see **Table 3**). Therefore, PET-based PSMA evaluation may provide interesting information for prognostication such as other first PET-based prognostic parameters in neuro-oncology have already shown to provide additional information in the clinical context (53–55). However it has to be noted, that the survival estimations in this study did not reach statistical significance (p = 0.066), which can probably be attributed to the small number of patients included. The study is further limited by its retrospective study design. In addition, according to the study design, only patients whose clinical course allowed for a second tumor resection were included, which could possibly imply a selection bias with regard to survival analysis. Furthermore, the study cohort is characterized by moderate variability with respect to risk factors such as age at initial diagnosis (range 25-74 years) and number of cycles of adjuvant temozolomide (range 1-12 months). Given the small number of cases, this may be seen as a limitation regarding survival analyses. However, with regard to the detection of PSMA expression per se, a broader study population may on the other hand also be seen as an advantage, and highly selected patient collectives in neuro-oncology have already been discussed as a narrowing that does not pay sufficient attention to the entire affected patient population (56). Therefore, the inverse correlation of PSMA expression and survival in glioblastoma patients found in the current study has eventually to be interpreted with caution and prospective studies with larger cohorts of glioblastoma patients are warranted. Thus, further investigations on PET imaging of PSMA expression are necessary in order to determine validated cut-off values for prognostication in glioblastoma.

## Conclusion

PSMA is a promising target for theranostic approaches in glioblastoma. PSMA is expressed in glioblastoma both at initial diagnosis and at recurrence after multimodal therapy, however to a variable extent. A high level of vascular PSMA expression following multimodal therapy as well as an increasing PSMA expression over time seem to be associated with a poor prognosis. The results of the current study foster the knowledge on PSMA expression in glioblastoma and warrant further studies in the field of PSMA-directed treatment concepts in neuro-oncology. Translating these results into PSMA PET imaging studies would be a desirable and congruous next step.

## Data Availability Statement

The original contributions presented in the study are included in the article/supplementary material. Further inquiries can be directed to the corresponding author.

## Ethics Statement

The studies involving human participants were reviewed and approved by Ethikkommission bei der Medizinischen Fakultät der LMU München. Written informed consent for participation was not required for this study in accordance with the national legislation and the institutional requirements.

## Author Contributions

BS conceived and designed the study with the help of J-CT and NA. AB, J-CT, and BS provided the tissue specimens and the clinical data. VR and JH provided the stainings and the genetic data. AH, AB, VR, and FL-S performed IHC analyses. KS, JS, and JH provided advices for IHC analyses. AH, MK, MU, LM, PB, and NA performed PET imaging and PET analyses. AH and BS performed analyses of clinical data. AH wrote the manuscript under the guidance of BS, NA, and J-CT and with further input from all co-authors. All authors contributed to the article and approved the submitted version.

## Funding

This project includes collaboration within the CRC 824 (research area B2 and Z2) of the German Research Foundation (DFG), project number 68647618. NA thanks the Else Kröner Fresenius-Stiftung for the support of her research.

## Conflict of Interest

The authors declare that the research was conducted in the absence of any commercial or financial relationships that could be construed as a potential conflict of interest.

## Abbreviations

^18^F, fluorine-18; ^18^F-DCFPyL, 2-(3-(1-carboxy-5-[(6-[^18^F]fluoro-pyridine-3-carbonyl)-amino]-pentyl)-ureido)-pentanedioic acid; ^68^Ga, gallium-68; ^89^Zr, zirconium-89; ^89^Zr-Df-IAB2M, 89Zr-desferrioxamine-IAB2M; ^177^Lu, lutetium-177; AZ, Arizona; CA, California; CI, confidence interval; CT, computed tomography; EORTC, European Organisation for Research and Treatment of Cancer; FFPE, formalin-fixed and paraffin-embedded; FOLH1, folate hydrolase 1; GCPII, glutamate carboxypeptidase II; H&E, hematoxilin- and eosin; IDH, isocitrate dehydrogenase; IHC, immunohistochemistry; IRS, immunoreactive score; KPS, Karnofsky performance status scale; ldCT, low-dose computed tomography; LMU, Ludwig-Maximilians-Universität; MBq, megabecquerel; MRI, magnetic resonance imaging; MGMT, O-6-methylguanine-DNA methyltransferase; NAAG, N-acetylaspartylglutamic acid; NAALADase I, N-acetyl-l-aspartyl-l-glutamate peptidase I; NCIC, National Cancer Institute of Canada; PET, positron emission tomography; PRRT, peptide receptor radionuclide therapy; PSMA, prostate-specific membrane antigen; USA, United States of America; WHO, World Health Organization.
